# Galunisertib suppresses the staminal phenotype in hepatocellular carcinoma by modulating CD44 expression

**DOI:** 10.1038/s41419-018-0384-5

**Published:** 2018-03-07

**Authors:** Bhavna Rani, Andrea Malfettone, Francesco Dituri, Jitka Soukupova, Luigi Lupo, Serena Mancarella, Isabel Fabregat, Gianluigi Giannelli

**Affiliations:** 1 School of Medicine, University of Bari, Bari, Italy; 2 Oncobell Program, Bellvitge Biomedical Research Institute (IDIBELL), L’Hospitalet, Barcelona, Spain; 3 National Institute of Gastroenterology “S. de Bellis” Research Hospital, Castellana Grotte, Bari Italy; 4 Department of Physiological Sciences, Faculty of Medicine and Health Sciences, University of Barcelona, Barcelona, Spain

## Abstract

Cancer stem cells (CSCs) niche in the tumor microenvironment is responsible for cancer recurrence and therapy failure. To better understand its molecular and biological involvement in hepatocellular carcinoma (HCC) progression, one can design more effective therapies and tailored then to individual patients. While sorafenib is currently the only approved drug for first-line treatment of advanced stage HCC, its role in modulating the CSC niche is estimated to be small. By contrast, transforming growth factor (TGF)-*β* pathway seems to influence the CSC and thus may impact hallmarks of HCC, such as liver fibrosis, cirrhosis, and tumor progression. Therefore, blocking this pathway may offer an appealing and druggable target. In our study, we have used galunisertib (LY2157299), a selective ATP-mimetic inhibitor of TGF-β receptor I (TGFβI/ALK5) activation, currently under clinical investigation in HCC patients. Because the drug resistance is mainly mediated by CSCs, we tested the effects of galunisertib on stemness phenotype in HCC cells to determine whether TGF-β signaling modulates CSC niche and drug resistance. Galunisertib modulated the expression of stemness-related genes only in the invasive (HLE and HLF) HCC cells inducing a decreased expression of CD44 and THY1. Furthermore, galunisertib also reduced the stemness-related functions of invasive HCC cells decreasing the formation of colonies, liver spheroids and invasive growth ability. Interestingly, CD44 loss of function mimicked the galunisertib effects on HCC stemness-related functions. Galunisertib treatment also reduced the expression of stemness-related genes in ex vivo human HCC specimens. Our observations are the first evidence that galunisertib effectiveness overcomes stemness-derived aggressiveness via decreased expression CD44 and THY1.

## Introduction

Hepatocellular carcinoma (HCC) is the most common form of primary liver cancer. It is the fifth most common cancer globally and the third most common cause of cancer-related deaths^1^. HCC is a complex ailment associated with numerous risk factors, thus making it difficult to treat. Patients with underlying cirrhosis or chronic liver diseases are at a high risk of developing HCC. Since symptoms defining HCC are usually absent, patients typically manifest the symptoms of cirrhotic liver patients^2^. Various insults to liver such as exposure to the toxic substances (aflatoxins), infection with hepatitis B/C viruses, or abusive use of alcohol elicit the immune response in the liver, which results in the inflammation via the activation of Kupffer cells and hepatic stellate cells and could also result in necrosis^3^. During this time, different factors, particularly transforming growth factor (TGF)-*β*^4,5^ are involved in inducing the wound-healing response. Mutations or alterations in this controlled mechanism could lead to the formation of fibrosis and later on cirrhosis^6^. Cirrhosis is the advanced stage of fibrosis and is characterized by the distortion of the liver parenchyma associated with septate and nodule formation, altered blood flow, and risk of failure^7^.

Furthermore, tissue microenvironment plays a major role in this process of HCC^8,9^. Interaction of a variety of cell types in the tumor stroma with extracellular matrix components leads to the attainment of an abnormal phenotype that promotes tumor formation. Hypoxia enhances progression and metastasis of HCC. Activation of epithelial–mesenchymal transition (EMT), oxidative stress, and inflammation also play major roles in tumor progression^10^. Furthermore, cells with ‘stem-like’ features, termed cancer stem cells (CSCs), are also present in the liver during HCC that promotes tumor growth^11^. Indeed, a better understanding of the cell types involved in the initiation and progression of liver cancer may help to better understand molecular mechanisms of carcinogenesis and therapeutic options.

TGF-β is a multifunctional cytokine that controls proliferation, cellular differentiation, adhesion, migration, apoptosis and other functions in most cells^12^. TGF-β acts as a tumor suppressor in normal tissues, but once tumor cells acquire mechanisms to overcome its suppressor effects, it causes tumor progression^13^. The switch from tumor suppressor to oncogenic is not well understood yet, but intrinsic and extrinsic factors seem to play important roles. The loss of cell polarity, acquisition of motile properties, and a mesenchymal phenotype during EMT are considered critical intrinsic changes of the tumor cells^14^. A pro-tumorigenic potential of TGF-β signaling is also mediated by extrinsic factors originating from the tumor microenvironment, such as angiogenesis, inflammation, and fibroblast activation. In addition to changes in the tumor tissue, modifications in the TGF-β signaling pathway can also contribute to tumor growth. Hence, TGF-β plays a vital role in the molecular pathogenesis of HCC; TGF-β targeting could provide new insights in the clinical setting^4^. Several small-molecule inhibitors of TGF-β receptor I/II (TGFβRI/II/ALK5) have been developed, of which three are currently in clinical development^15^. Galunisertib (LY2157299), a selective ATP-mimetic inhibitor of TGFβRI/ALK5, is the only TGF-β pathway inhibitor currently under clinical investigation in HCC patients (NCT01246986). Recently, galunisertib has been shown to efficiently inhibit the expression of p-Smad2 as well as invasion, but not proliferation, in three HCC models in vitro^16^.

Keeping this in mind, here we aimed to use galunisertib to understand its role in modulating the expression of stemness-related genes and its effect on the clonogenicity, spheroid formation, and invasiveness of HCC. This work was designed to help develop a strategy on how to best select patients for drugs that have been implicated to alter the CSC niche or its associated biology.

## Results

### Invasive and non-invasive HCC cells express a different stemness-related gene profile

**Fig. 1:**
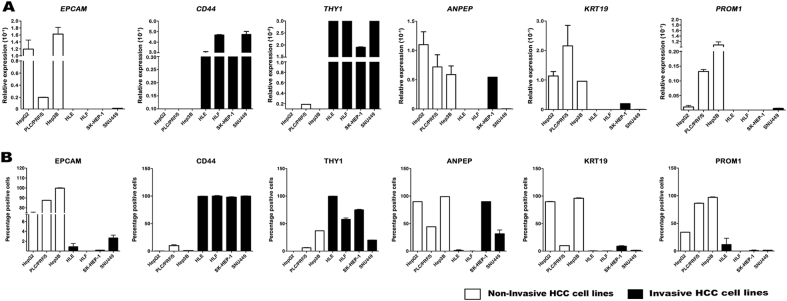
Invasive and non-invasive HCC cells show a distinctive expression pattern for stemness-related markers. **a** Relative expression of indicated stemness-related genes analyzed by qRT-PCR. Differences in the expression between non-invasive (white bars) and invasive (black bars) HCC cells were highly significant (*P* < 0.001) for all stemness-related genes and *P* < 0.005 for *PROM1*. **b** Flow cytometry analyses of indicated stemness-related proteins expressed on the cell surface. Results are expressed as % of positive cells. Mean ± SEM (*N* at least 3)

Basal mRNA expression level of *epithelial cell adhesion molecule* (*EPCAM*), *CD44*, *THY1* (also known as CD90), *alanyl aminopeptidase membrane* (*ANPEP*, also known as CD13*)*, *Keratin 19* (*KRT19),* and *prominin-1 (PROM1*, also known as CD133) was quantified in seven different HCC cell lines. Two different phenotypes of HCC cells were distinguished as non-invasive (also epithelial, namely HepG2, PLC/PRF/5, and Hep3B) and invasive (also mesenchymal, HLE, HLF, SK-HEP-1, and SNU449). The different phenotype of cells determines also differences in stemness-related gene profile. Non-invasive HCC cells expressed *EPCAM*, *ANPEP*, *KRT19*, and *PROM1*, whereas invasive HCC cells expressed more mesenchymal markers such as *CD44* and *THY1* (Fig. 1a). Differences in the expression of *EPCAM*, *CD44*, *THY1*, *ANPEP*, *KRT19*, and *PROM1* between non-invasive and invasive HCC cells were highly significant (*P* < 0.001 for all stemness-related genes and *P* < 0.005 for *PROM1*). Flow cytometry analyses confirmed the differential transcriptional pattern between the two groups of cells (Fig. 1b). The cell surface stemness-related EPCAM, ANPEP, and PROM1 were prevalent in non-invasive cells, along with the internal stemness-related KRT19, while CD44 and THY1 are dominant in the invasive HCC cells (Fig. 1b).

### Galunisertib modulates the stemness-gene profile of invasive HCC cells

**Fig. 2:**
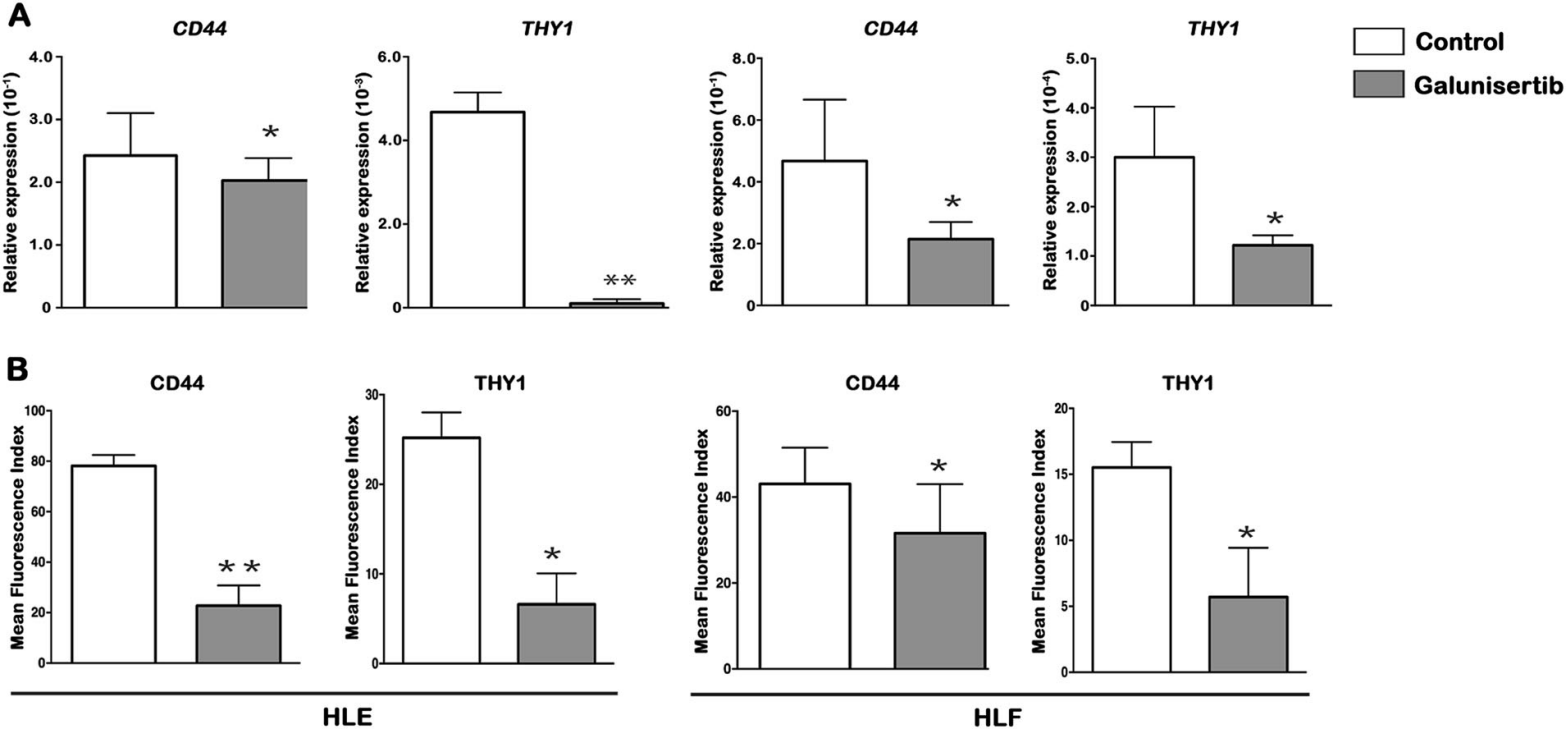
Galunisertib modulates the stemness-gene profile of invasive HCC cells. **a** Relative expression of *CD44* and *THY1* by qRT-PCR. **b** Flow cytometry analysis of CD44 and THY1 expressed on the cell surface. Results are expressed as MFI. Mean ± SEM (*N* at least 3). *P < 0.05; **P < 0.001

**Fig. 3:**
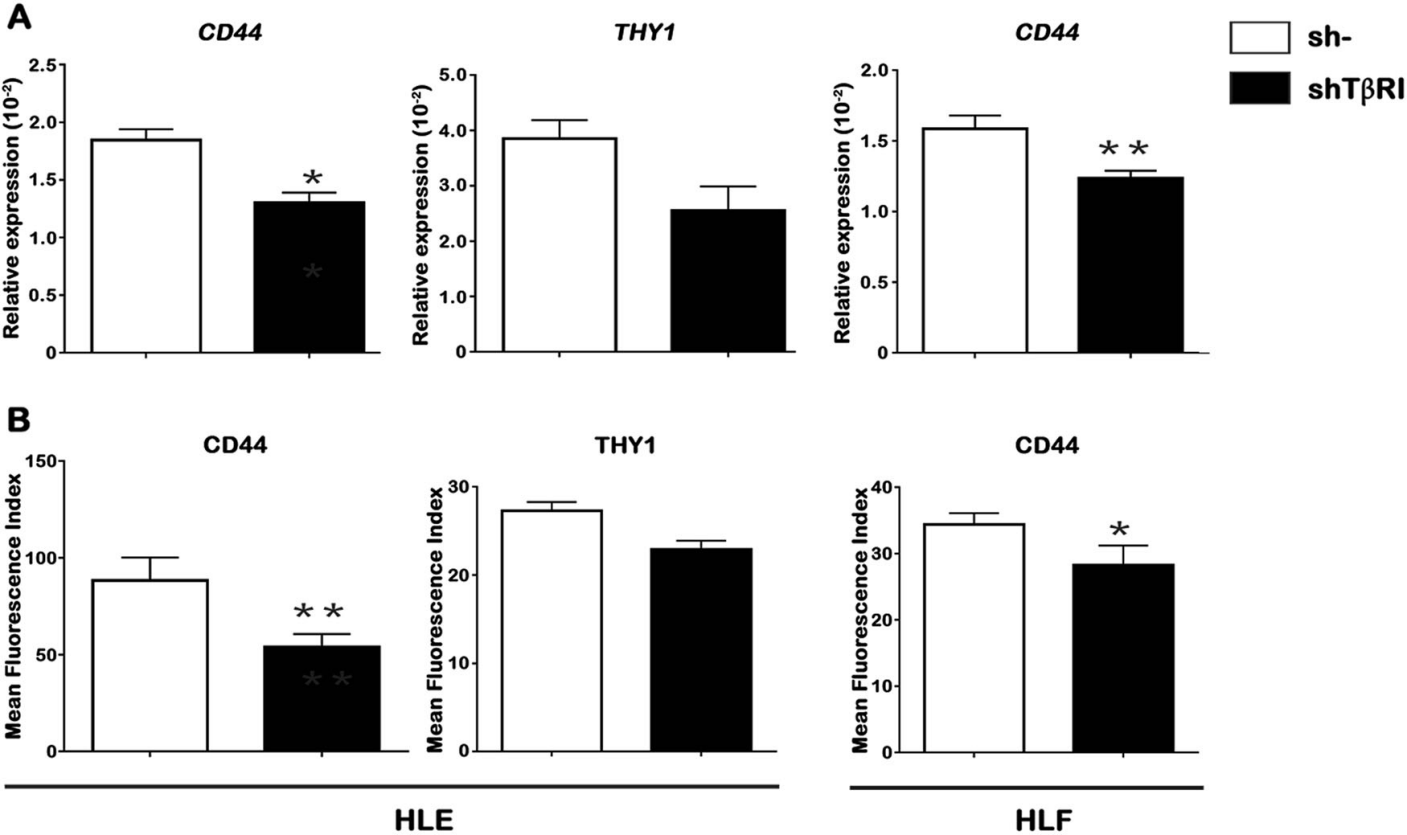
Effect of stable TGFβR1 knockdown on mesenchymal stemness-related gene expression in invasive HCC cells. **a** Relative expression of *CD44* and *THY1* genes analyzed by qRT-PCR. **b** Flow cytometry analysis of CD44 and THY1 markers expressed on the cell surface. Results are expressed as MFI. Mean ± SEM (*N* at least 3). *P < 0.05; **P < 0.001

To test the hypothesis that galunisertib-mediated blocking of TGFβRI affects stemness-gene profiles of invasive HCC cells, we challenged them with galunisertib. A long-term treatment with galunisertib significantly reduced the expression of *CD44* and *THY1* in HLE and HLF invasive cells (Fig. 2a). Furthermore, flow cytometry analyses confirmed that galunisertib treatment notably shifted the mean fluorescence index of CD44 and THY1 as compared to untreated cells (Fig. 2b). Altogether, data indicate that effective inhibition of TGFβRI by galunisertib reduces the stemness-gene profile of invasive HCC cells, thus also demonstrating the potential role of TGF-β in regulating the stemness of HCC. To reinforce the hypothesis that the TGF-β pathway regulates stemness-related gene expression in HCC cells, we targeted to knockdown the TGFβRI with specific short hairpin RNA (shRNA) in the invasive HCC cells. Down-regulation of TGFβRI induced acquisition of a more epithelial-like morphology in HLE and HLF cells (Supplementary Figure 1A). Efficiency in the reduction of CD44 expression following its targeting knockdown was analyzed by flow cytometry: presence of TGFβRI on the plasma membrane (purple peaks in Supplementary Figure 1B), compared to cells transfected with an unspecific shRNA (sh-: blue peaks) and unstained cells used as an internal control (Blank: black peaks). Western blots also demonstrated the decrease in TGFβRI levels, which correlated with strong inhibition in the signaling, analyzed as SMAD2 phosphorylation (Supplementary Fig. 1C). Interestingly, we observed that targeting knockdown of TGFβRI exerted similar effects as galunisertib treatment on decreasing the expression levels of *CD44*, analyzed by real-time PCR (Fig. 3a) or flow cytometry (Fig. 3b).

### Galunisertib reduces clonogenicity and liver spheroid formation ability of HCC invasive cells

**Fig. 4:**
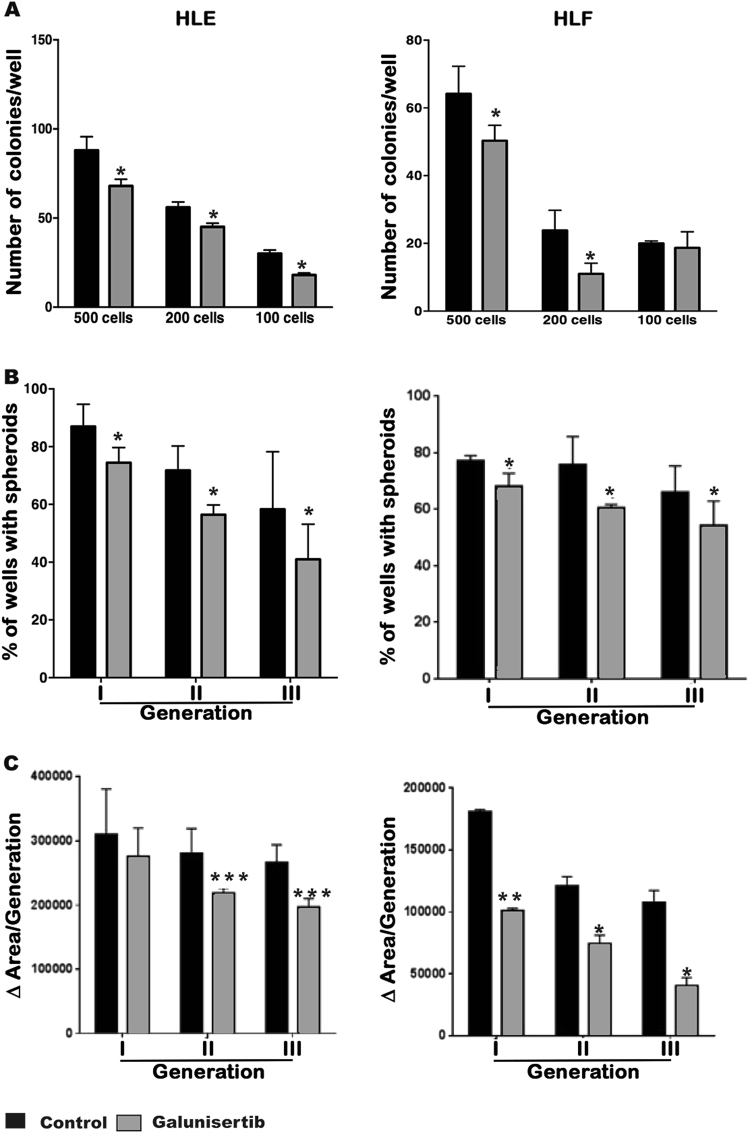
Galunisertib reduces clonogenicity and liver spheroid formation ability of HCC invasive cells. **a** Colony formation assay. After galunisertib treatment, HLE (left panel) and HLF (right panel) cells were serially diluted into 500, 200, and 100 cells and cultured for 12 days. Data represent quantification of the number of colonies per well. **b** Ability of growing in spheroids in the absence of attachment. The % of wells with spheroids during first, second, and third cell generation is shown. **c** Variation on the area of liver spheroids during first, second, and third cell generation is shown. Mean ± SEM (*N* at least 3). *P < 0.05; **P < 0.001; ***P < 0.0001

To investigate the effects of the galunisertib regulation on stemness-gene expression in terms of functionality, we measured the clonogenic capacity (the ability of single cells to undergo unlimited division and to form colonies) and the liver spheroid formation in HLE and HLF cells after galunisertib treatment (Fig. 4). Consistently with our previously described results, galunisertib reduced the formation of colonies at various cell densities, the percentage of liver sphere formation, as well as the area of the liver spheres, which might be also due to the galunisertib-mediated inhibition of cell proliferation.

### Galunisertib reduces the invasive growth capacity of HCC cells

**Fig. 5:**
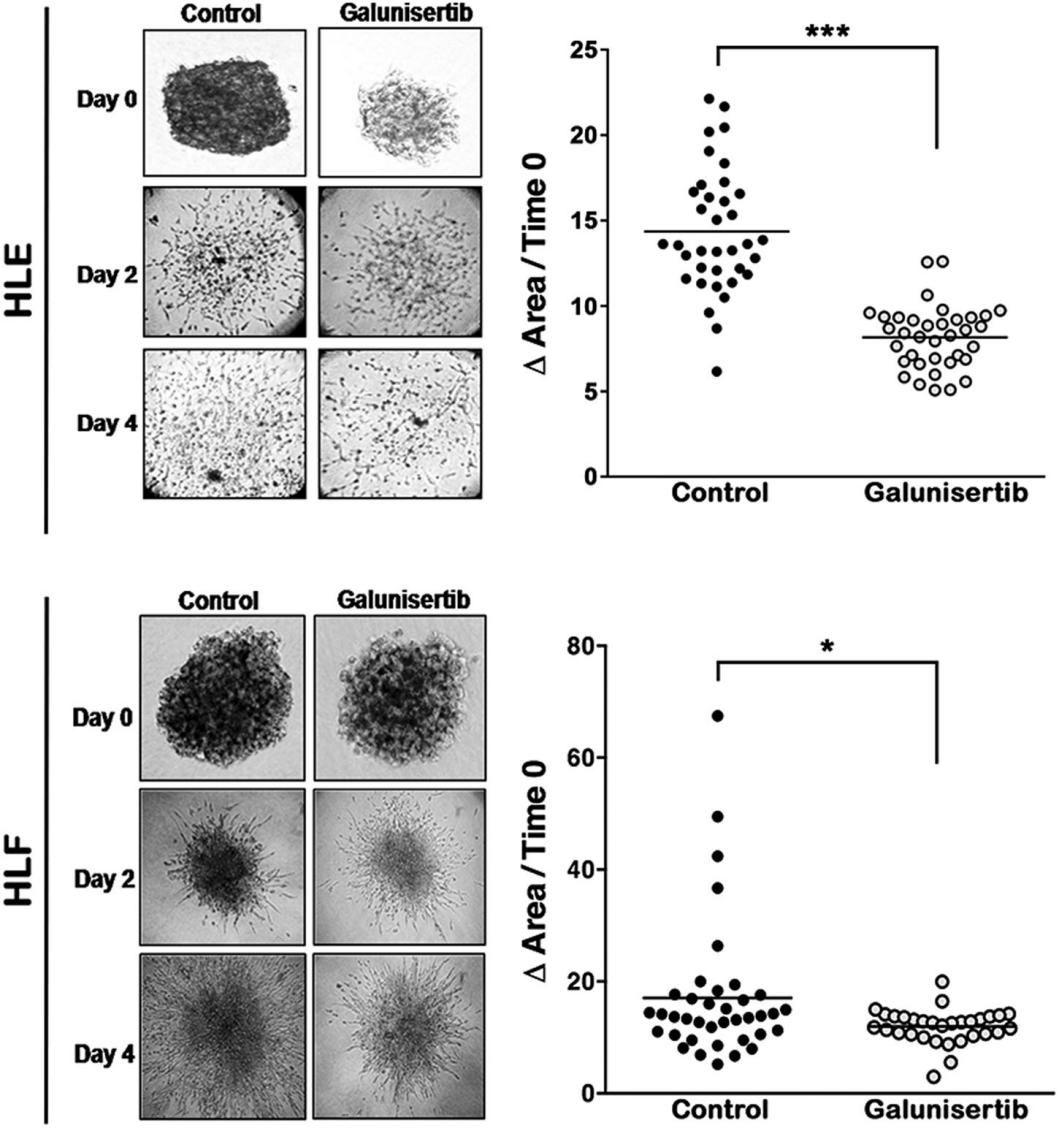
Galunisertib reduces the invasive growth capacity of HCC cells. Invasive growth assay in 3-D culture. Representative images of spheroids obtained with HLE (**a**) and HLF (**b**) cells are shown in the left panel. The quantification of invasive growth capacity show (right panel) was calculated as the increase of the area occupied by the spheroids between day 0 and day 4. The line in the dot-plot graph represents mean value

The three-dimensional (3-D) tumor spheroid invasion assay is a vastly robust technique used to form 3-D spheroids using the hanging drop technique. To evaluate the effect of galunisertib on the functionality of the invasive HCC cells (HLE and HLF), we pretreated the cells for 48 h in the presence or absence (control, dimethyl sulfoxide [DMSO]) of galunisertib. The generated 3-D spheroids were embedded into the collagen I matrix to allow the invasive growth of the cells. As reported in Fig. 5, galunisertib decreased the invasive capacity of HLE and HLF cells respect to controls.

### Stable knockdown of CD44 mimicked the effects of galunisertib on HCC stemness properties

**Fig. 6:**
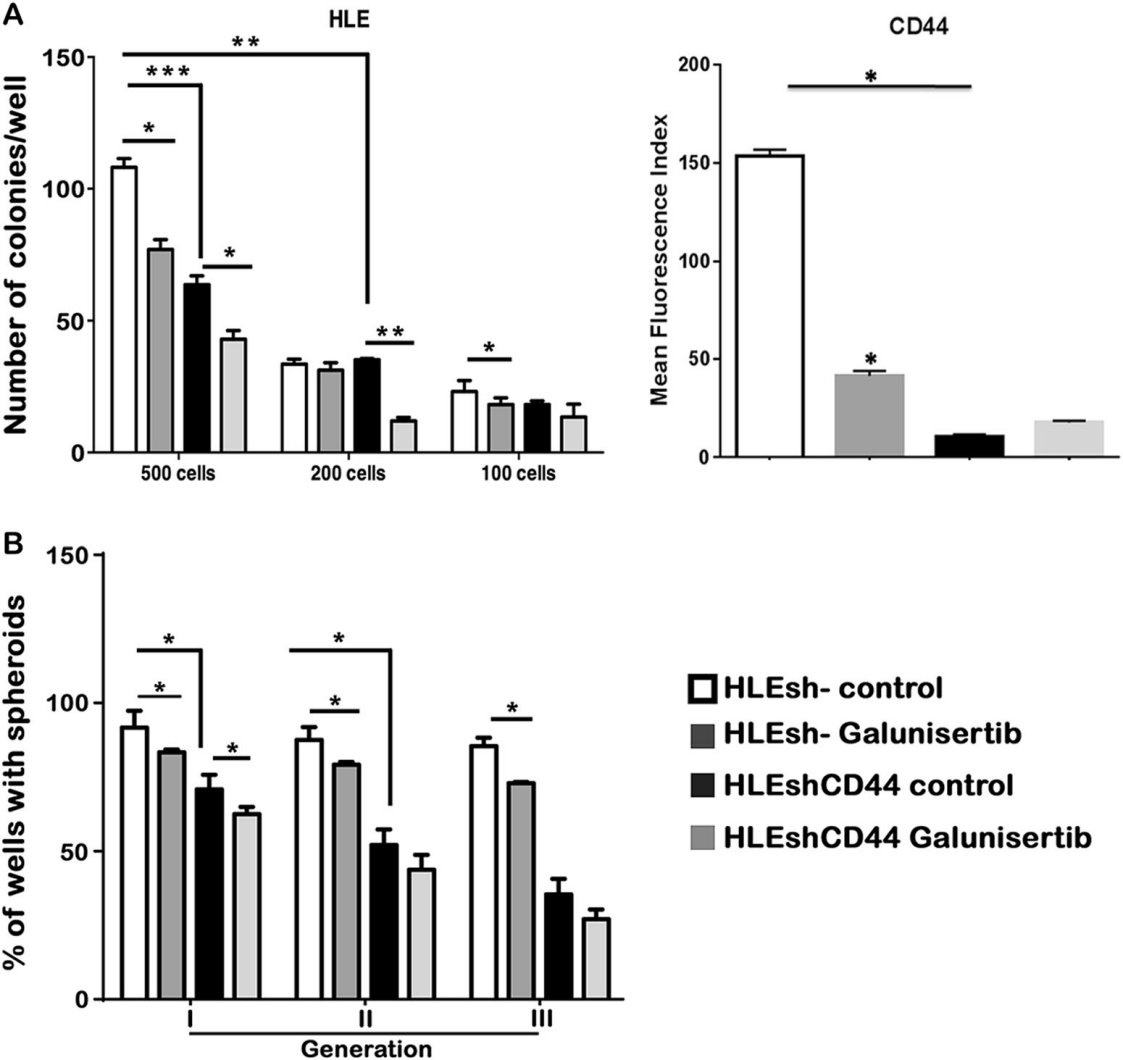
Stable knockdown of CD44 mimicked the effects of galunisertib on HCC stemness properties. **a** Colony formation assay. After galunisertib treatment, HLEsh− and HLEshCD44 cells were serially diluted into 500, 200, and 100 cells and cultured for 12 days. Data represent quantification of the number of colonies per well. Flow cytometry analyses of CD44 expressed on the cell surface are shown (right panel). Results are expressed as MFI. **b** Ability to grow in spheroids in the absence of attachment. The % of wells with spheroids during first, second and third cell generation is shown. Mean ± SEM (*N* at least 3). *P < 0.05; **P < 0.001; ***P < 0.0001

**Fig. 7:**
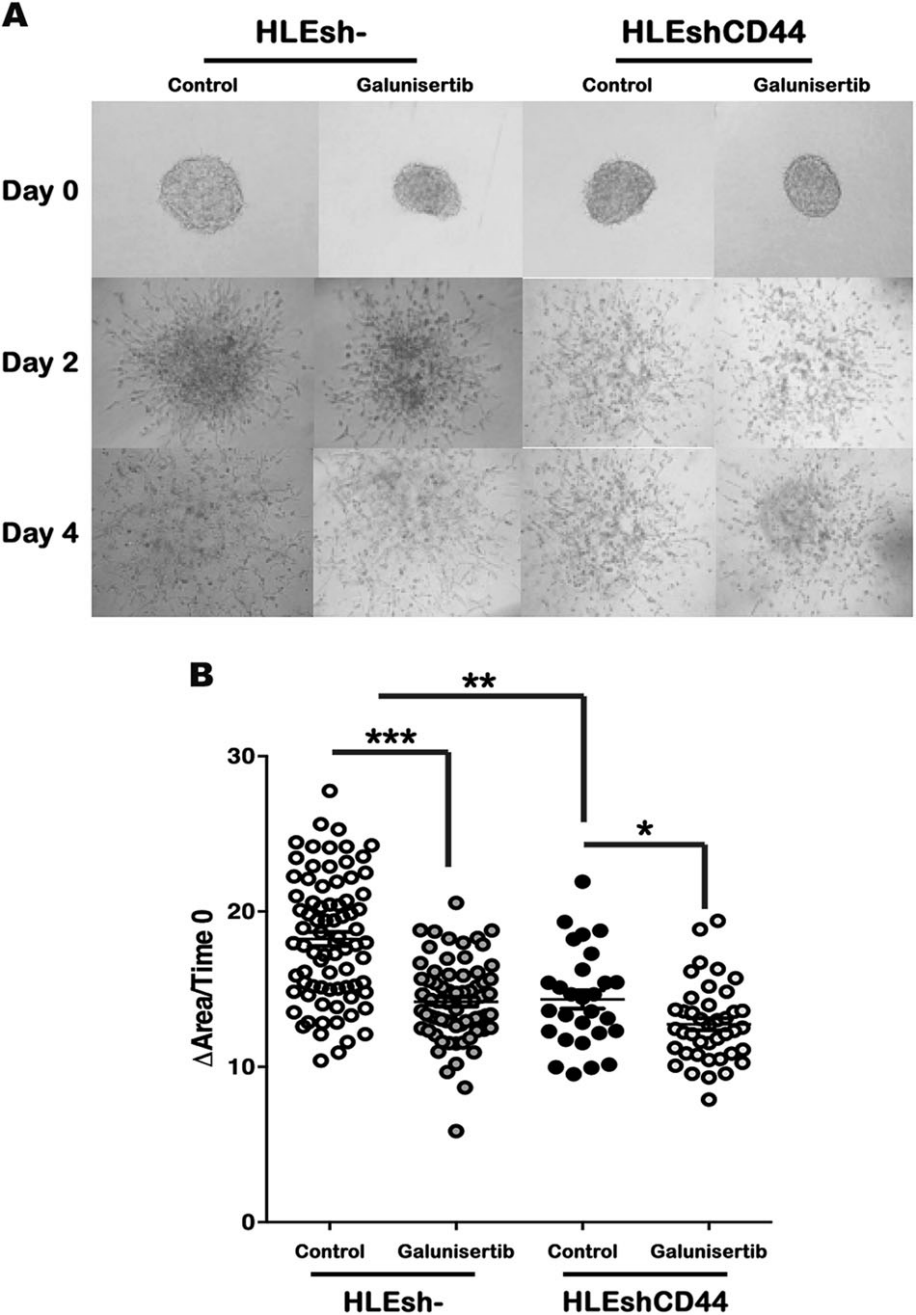
Targeting knockdown of CD44 mimicked the effects of galunisertib on invasive properties of HCC cells. **a** Invasive growth assay in 3-D culture. Representative images of spheroids are shown. **b** Quantification of invasive growth capacity in 3-D culture. The increase of the area occupied by the spheroids between day 0 and day 4 was calculated. The line in the dot-plot graph represents mean value. *P < 0.05; **P < 0.001; ***P < 0.0001

To explain galunisertib effectiveness on HCC cell stemness and invasiveness we tested the hypothesis that the modulation of CD44 expression mediates such effects. We induced CD44 knockdown in HLE cells through shRNA. The capacity to form colonies at low dilutions in the HLEshCD44 cells was significantly lower than in control cells (Fig. 6a). HLEshCD44 cells also formed a lower number of liver spheroids as compared to the HLEsh− controls (Fig. 6b). These results indicate the relevance of CD44 expression on the stemness-related functional properties of HCC cells. Interestingly, when we analyzed the additional effect of galunisertib on these properties in HLEshCD44 cells, we observed a further reduction in their clonogenic capacity, but barely effect on the ability of cells to form spheroid, when compared with the corresponding untreated HLEshCD44 cells, suggesting that the decrease in CD44 expression may be the main molecular mechanism undergone by galunisertib to reduce spheroid formation capacity of the HCC cells. Knockdown of CD44 also produced a decrease in the invasive ability of HLE cells (Fig. 7), similar to galunisertib treatment. In parallel to the results observed on the stemness-related properties, treatment of galunisertib in HLEshCD44 cells showed only a slight additional effects on their invasive capacity. Altogether, these results indicate that targeting knockdown of CD44 mimicked most of the effects of galunisertib on CSCs and invasive properties in HLE cells.

### Galunisertib reduces the expression of stemness-related genes in ex vivo HCC patients’ samples

**Fig. 8:**
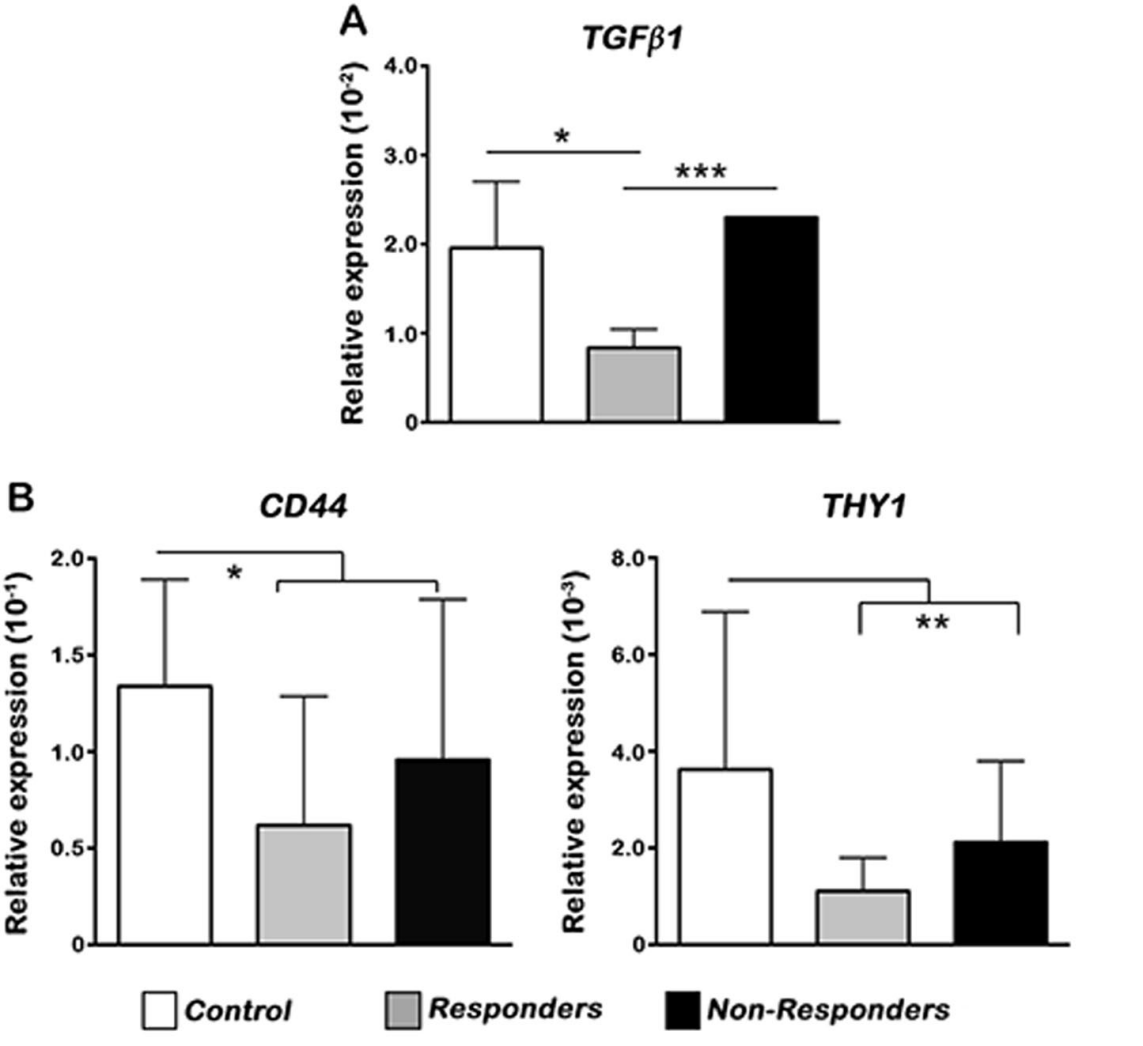
Galunisertib reduces the expression of stemness-related genes in ex vivo HCC patients’ samples. **a** Relative expression of *TGFβ1* analyzed by qRT-PCR following treatment with galunisertib in ex vivo tumor samples from HCC patients. **b** Relative expression of *CD44* and *THY1* analyzed by qRT-PCR. Mean ± SEM (number of patients = 24, each experiment technically performed in triplicate). *P < 0.05; **P < 0.001

To investigate the relevance in vivo, we quantified the expression of *TGFβ1* and stemness-related genes following treatment with galunisertib in ex vivo tumor samples from 24 HCC patients. Tumor samples were categorized as responders (13) and non-responders (11), based on *TGFβ1* expression after galunisertib treatment in ex vivo experimental model. In the responders cohort, galunisertib treatment reduced the expression of *CD44* and *THY1* genes as compared to the controls (Fig. 8). These results suggest that *TGFβ1* expression in HCC patients might play a significant role in maintaining CSC niche during HCC and, subsequently, the aggressiveness of this malignancy, while galunisertib is effectively reducing the stemness-related gene expression in HCC patients’ samples. These data support our in vitro findings and might serve as a strong evidence of galunisertib use as a potential targeted therapy against HCC.

## Discussion

Herein, we studied a panel of staminal biomarkers in seven different epithelial and mesenchymal HCC cell lines with different epithelial–mesenchymal characteristics and different response to TGF-β as suppressor or pro-tumorigenic^17–19^. Our results indicate that HCC cells exhibited a distinctive profile of stemness-related genes according to their TGF-β-dependent signature: early (epithelial, responding to the suppressor effects of TGF-β) or late (mesenchymal with autocrine production of TGF-β and unresponsive to its suppressor effects). In particular, the non-invasive, epithelial “early” TGF-β signature HCC cells express EPCAM, ANPEP, KRT19 and PROM1, whereas the invasive, mesenchymal “late” TGF-β signature HCC cells express CD44 and THY1. Endothelial SK-HEP-1 cells express ANPEP, previously described as a marker of monocytes and endothelial cells^20^. Our study differs from others, since we examined a broad panel of stemness-related genes in various invasive and non-invasive HCC cells. TGF-β has been recently reported to act as a mediator of the mesenchymal phenotype observed in some HCC cell lines^18,21^ and HCC tissues^22^, which bestows these cells with a higher migratory and invasive capabilities and apoptosis resistance. In our study, the knockdown of TGFβRI in HLE and HLF cells reduces the expression of mesenchymal markers, in particular, CD44 and THY1, both at mRNA and protein levels. Knockdown of TGFβRI exerts similar effects as observed by inhibiting TGFβRI with its specific inhibitor galunisertib. We found that galunisertib reduces the expression of CD44 and THY1 in HLE and HLF cells at both mRNA and protein levels. Altogether, these results indicate that TGF-β mediates the expression of CD44 and THY1 in invasive HCC cells. CD44 is a cell surface glycoprotein that functions as a receptor for hyaluronic acid^23^, is involved in cell–cell adhesion and migration, and has been showed to be associated with tumor cell invasion and migration in liver cancer^24,25^. We recently found that when TGF-β induces EMT in HCC cells, a switch in the expression of stemness-related genes is observed, concomitant with up-regulation of CD44, and their stemness potential and migratory/invasive capacity are enhanced^26,27^. Here we show that galunisertib reduces the expression of CD44, affecting the ability of invasive HCC cells to form colonies, liver spheroids, and 3-D invasion. Moreover, galunisertib exhibited anti-proliferative activity in ex vivo models representing a more physiological model, signifying that inhibition of TGF-β can exert anti-tumoral effects, which might be mediated by the tumor microenvironment^28,29^. Its use under clinical phase II trial study (Eli Lilly) is allowing the design of a road map for personalizing cancer therapy in HCC by using molecular pattern diagnostics^30^. This is an important first evidence-based study presented the efficacy of galunisertib treatment in HCC, where it modulates the expression of stemness genes and hence providing the proof to support its use in currently ongoing clinical trials.

Recent evidence has reported that CD44 promotes tumor invasion and metastasis in multiple cancer types including colon, prostate, bladder, breast, and liver cancers^31–33^. The proposed mechanism is that CD44 inhibits the membrane-located E-cadherin and *β*-catenin, resulting in nuclear translocation of *β*-catenin and transcriptional activation of genes related to cell invasion and migration^32,33^. This is consistent with our new findings, where the silencing of CD44 inhibits the colony formation, liver spheroids formation and maintenance, as well as invasive growth ability in HLE cells, indicating the potential role of CD44 in liver pathogenesis. The attenuation or even loss in the response to galunisertib in CD44 knockdown cells indicate that down-regulation of *CD44* by galunisertib may be one of the relevant mechanisms undergone by this drug to mediate its effects on stemness and invasion in HCC cells.

Our ex vivo assays of human HCC patient samples supports the in vitro data. *TGFβ1* signatures were different in different HCC samples before or after treatment with galunisertib. The expression of *CD44* and *THY1* were reduced in responders as compared to the non-responders. Previous research has already shown that TGF-β is upregulated in HCC and plays an important role in the HCC progression. Also, it has been indicated that CD44s plays a critical role in the TGF-β-mediated mesenchymal phenotype and is associated with poor prognosis of HCC patients^25^ and also that THY1 and CD44 act as a potential biomarker for HCC^34^. Moreover, our data emphasized the potential use of galunisertib-mediated regression of HCC, particularly, to decrease the expression of stemness genes and associated decrease of stemness-related abilities.

In summary, we have shown for the first time the effect of galunisertib on the expression of stemness genes in HCC. Our study strongly supports the promising beneficial use of galunisertib clinically, as this drug reduces the clonogenic capacity, liver spheroid formation, and invasive ability of HCC cells in vitro. Furthermore, our results extend previous data indicating that CD44 might be under the control of *TGFβ1* signaling, contributing to the more aggressive phenotype of HCC. Knocking down of CD44 proved the essential role of CD44 in the formation of colonies, liver spheroid, and invasive ability in vitro, mimicking the results obtained from the inhibition of *TGFβ1* by galunisertib, where the drug reduced the expression of CD44 and hence decreased the functionality of HCC cells. Our in vitro data are supported by the ex vivo study on HCC patient samples, where we have shown that galunisertib effectively reduces the expression of *TGFβ1* in responders, but not in non-responder patients. Moreover, we have shown that galunisertib efficiently reduced the expression of stemness genes in responders. These experimental studies would be relevant in accessing the efficacy of the galunisertib treatment, in order to improve therapeutic and management decisions in personalized medicine.

## Material and methods

### Cell lines and culture conditions

HepG2, PLC/PRF/5, Hep3B, SK-HEP-1 and SNU449 cells were obtained from ATCC (Manassas, VA, USA). HLE and HLF cells were from the Japanese Collection of Research Bioresources Cell Bank (JCRB Cell Bank, Japan). Cell lines were never used in the laboratory for longer than four months after receipt or resuscitation. Mycoplasma contamination was excluded in all the results presented in the manuscript. HepG2, PLC/PRF/5, Hep3B, and SK-HEP-1 cells were cultured in Dulbecco’s modified Eagle's medium (DMEM) with 4.5 g/L d-glucose. HLE, HLF, and SNU449 cells were cultured in Roswell Park Memorial Institute (RPMI) 1640 medium with l-glutamine, Phenol red. Both supplemented with 10% heat-inactivated fetal bovine serum (FBS) from Gibco^®^ (Life Technologies™, Paisley, UK), penicillin (100 U/mL), streptomycin (100 *μ*g/mL), and sodium bicarbonate (1.5 g/L). Trypsin 0.05%-EDTA 0.02% with Phenol red was purchased from EuroClone (Italy) and 4-(2-hydroxyethyl)-1-piperazineethanesulfonic acid) HEPES (20 nm) from Gibco^®^. Methyl cellulose and collagen IV solution were from Sigma-Aldrich (St. Louis, MO, USA). Matrigel matrix was from Corning (NY, USA). Cells were maintained at 37 °C in a humidified atmosphere and 5% CO_2_.

### Analysis of gene expression

A total of 100 ng RNA of HCC cell lines and 1 µg RNA from Human HCC tissues were used for first-strand complementary DNA (cDNA) synthesis for gene expression analysis. RT-PCR amplification was performed using CFX96 Real-Time system (C1000 Thermal cycler) (BioRad). Data were analyzed according to the delta-delta Ct method. Triplicate wells and biological triplicates were analyzed in each assay. Glyceraldehyde 3-phosphate dehydrogenase gene (*GAPDH*) was used as endogenous control. The following primers were obtained from Integrated DNA Technologies^®^ (Leuven, Belgium):

#### TGFβ1

Forward: 5′-CCG AGA AGC GGT ACC TGA AC-3′.

Reverse: 5′-GAG GTA TCG CCA GGA ATT GTT G-3′.

#### EPCAM

Forward: 5′-TCC AGA ACA ATG ATG GGC TTT-3′.

Reverse: 5′-TTG CAC TGC TTG GCC TTA AA-3′.

#### PROM1

Forward: 5′-ACA GCG ATC AAG GAG ACC AAA-3′.

Reverse: 5′-TTT GTT GGT GCA AGC TCT TCA-3′.

#### CD44

Forward: 5′-TGG AGA AAA ATG GTC GCT ACA G-3′.

Reverse: 5′-GGG CAA GGT GCT ATT GAA AGC-3′.

#### THY1

Forward: 5′-ACC TCT TCC TCT TCC CTG ACT TC-3′.

Reverse: 5′-GCC CAG TGT GCA GTC ATT AGC-3′.

#### ANPEP

Forward: 5′-CCG TCT ACT GCA ACG CTA TCG-3′.

Reverse: 5′-ATT GAC CAG TGT GGC ATT TCG-3′.

#### KRT19

Forward: 5′-AGG TCA GTG TGG AGG TGG ATT C-3′.

Reverse: 5′-GCT TCG CAT GTC ACT CAG GA-3′.

#### GAPDH

Forward: 5′-CAC CAT CTT CCA GGA GCG AG-3′.

Reverse: 5′-GAC TCC ACG ACG TAC TCA GC-3′.

### Drug assay

Respective cells were treated either with control (DMSO), 5 ng/mL of recombinant human *TGFβ1* (PeproTech, Germany) or galunisertib (10 µM) (Eli Lilly and Company, Indianapolis, IN, USA), and in combination of *TGFβ1 *+ galunisertib. On the other hand, invasive HCC cell lines were treated with 10 µM of galunisertib and control (DMSO). RNA was isolated after 48 h of respective treatments and gene expression was analyzed by qRT-PCR. Triplicate wells and biological triplicates were analyzed in each assay.

### Flow cytometry

Procedure was carried out as described previously^27^. A total of 1 × 10^6^ cells were incubated in FACS buffer (FBS + 0.1% bovine serum albumin) and respective fluorescence-conjugated antibodies. Flow cytometry was performed using Beckman Coulter Counter analyzer (Beckman and Coulter) and analysis was performed using Kaluza analysis software (Beckman and Coulter).

### Western blotting

Procedure was carried out as described previously^18^. Total protein extracts were obtained using a lysis buffer containing 30 mM Tris–HCl pH 7.5, 5 mM EDTA, 150 mM NaCl, 1% Triton X-100, 0.5% sodium deoxycolate, 0.1% SDS, and 10% glycerol (1 h at 4 °C; centrifugation at 13,000 rpm, 10 min, 4 °C). Protein concentration was measured with the BCA Protein Assay kit (Pierce). β-ACTIN is shown as a loading control. Antibodies were used at a 1:1000 dilution, except for β-ACTIN (1:3000). Images were processed with Adobe Photoshop CS5.

### Colony formation assay

Procedure was carried out as described previously^27^. Colony assay was performed to determine the ability of a “single cell” to grow into a “colony”. Cells were serially diluted into 500, 200, 100 cells and cultured in six-well plates for 12 days. Fresh pulse of galunisertib was given every third day, until the end of the experiment. Upon termination of the assay, the colonies were stained with crystal violet (0.2% in 2% ethanol) and colonies, which consist of at least 50 cells, were scored and counted using a stereomicroscope. The number of colonies was calculated using ImageJ software.

### Invasive growth assay

Procedure was carried out as described previously^27^. Cells were resuspended in low viscosity media in a concentration of 4 × 10^5^ cells/mL and suspended as hanging drops for 72 h at 37 °C–5% CO_2_, to allow them to cluster into compact sphere-like formation. Post 72 h, spheroids were embedded into Pure Col^®^ Type I Bovine collagen solution (3 mg/mL) (1.7 mg/mL in DMEM) and incubated in the presence of 10 µM of galunisertib added to 10% FBS-containing media at 37 °C–10% CO_2_. Phase contrast pictures were taken every 24 h, during 4 days. On second and fourth day, fresh pulse of medium containing 10 µM of galunisertib were added to each respective well. For quantification, increase of the area occupied by the spheroids between day 0 and day 4 was calculated using ImageJ software.

### Analysis of spheroid formation capacity under anchorage-independent conditions

Cells were seeded as hanging drop (1000 cells/drop) in aGravityPLUS™ plate. Tumor spheroids of the first generation were transferred into InSphero GravityTRAP^™^ plates and the number of spheroids per plate was counted under microscope. Spheres were later dissociated into single cell suspensions and serially passaged under the same initial culture conditions. The percentage of wells with spheroids counted during every generation.

### Ethical approval, HCC patients’ sample, collection, and ex vivo assays

Approval for the human HCC patient study was obtained from the local Ethics Committee of the Azienda Ospedaliero-Universitaria Policlinico, Bari (Apulia), Italy. Patients were given prior written informed consent to the use of their tissues. This study was performed in accordance with the Helsinki Declaration and informed written consent was obtained from all patients. Additionally, the data were analyzed anonymously. Surgical samples were obtained during HCC patient surgery at Policlinico, Bari, Italy. Twenty-four HCC tissues were processed into three parts and processed for routine histology, snap-frozen in liquid nitrogen and for the ex vivo study. The tumor tissue was processed into 2 mm size and cultured for 48 h in Iscove’s modified Dulbecco’s medium supplemented with 20% FBS. After 48 h, the tissues were snap- frozen and later RNA was isolated from the frozen HCC tissue. RNA was quantified and transcribed into cDNA and stemness-related gene expression was analyzed by qRT-PCR. Patient samples were classified as responders and non-responders based on their endogenous TGF-β expression level, which is compared with control conditions (without galunisertib).

### Targeted knockdown assays

Four different shRNA plasmids for TGFβRI and two for CD44 as well as unspecific shRNA were kindly provided by Isabel Fabregat. Cells selected for the experiments are those where maximal knockdown of TGFβ was observed, as previously described^26,27^.

### Statistical analyses

Statistical analyses for all experiments were performed using two-tailed Student *t*-test and statistical significance was set at a *P-*value of <0.05. All data represent at least three experiments and are expressed as the mean ± standard error of the mean (SEM).

## Electronic supplementary material

Supplementary Figures
