# Supplementary material for: Galunisertib suppresses the staminal phenotype in hepatocellular carcinoma by modulating CD44 expression
Source: Cell Death Dis. 2018 Mar 7;9(3):373. doi: 10.1038/s41419-018-0384-5 (PMC5841307; doi:10.1038/s41419-018-0384-5)
Supplement: Supplementary file 1 — Supplementary Figures [file 41419_2018_384_MOESM1_ESM.pdf]

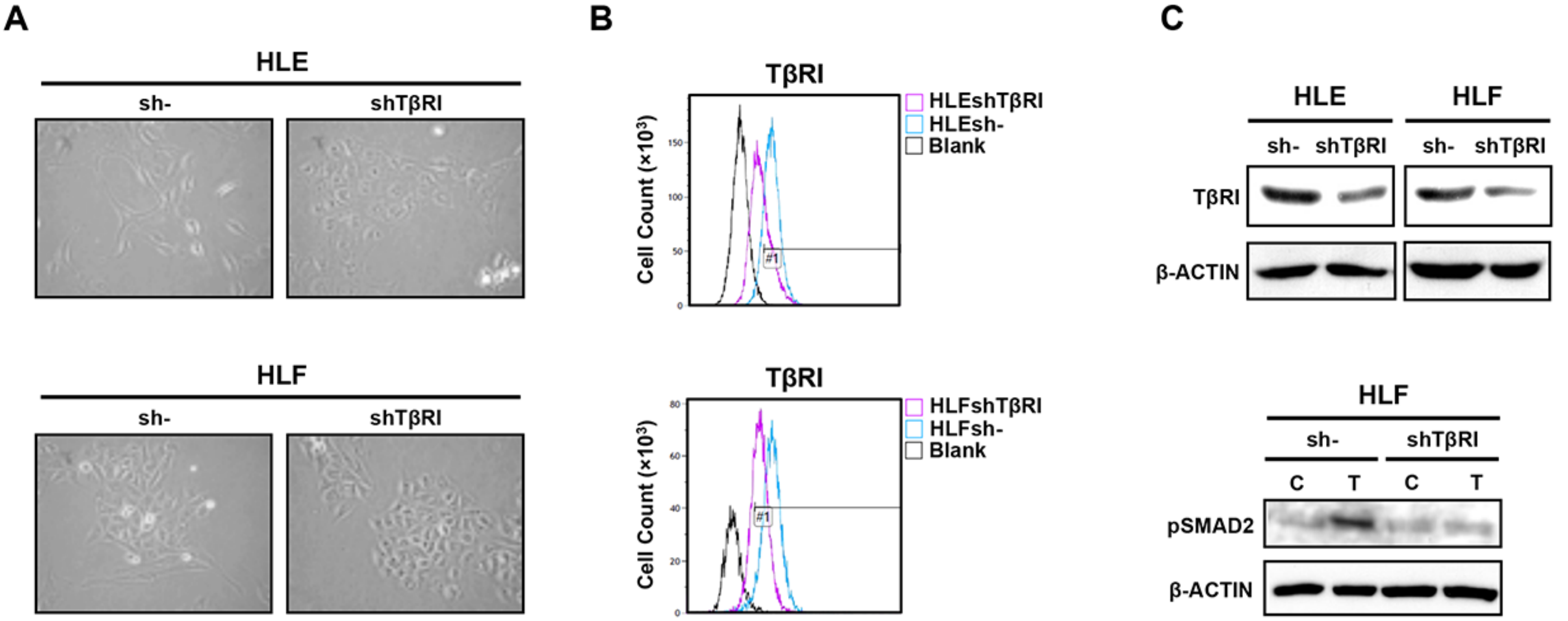

Supplementary Figure 1: Stable silencing of the TGF-β Receptor I (TβRI) in invasive HLE and HLF cells.
A. Phase contrast microscopy images of invasive HLE (*top*) and HLF (*bottom*) cells following targeting knockdown of the TβRI. **B.** Expression profiles for TβRI detected by flow cytometry analyses in HLE (*top*) and HLF (*bottom*) cells. Results are expressed as cell count of positive cells and a representative experiment is shown. **C.** Analysis by Western blotting assay of TβRI down regulation in HLE and HLF cells on basal conditions (*top*), and of p-SMAD2 down regulation on HLF cells following stimulation with TGF-β1 0.5ng/mL for 2 hours (*bottom*). A representative experiment is shown.
